# Addition of low dose hCG to rFSh benefits older women during ovarian stimulation for IVF

**DOI:** 10.1186/1477-7827-10-55

**Published:** 2012-08-06

**Authors:** Hala Gomaa, Robert F Casper, Navid Esfandiari, Paul Chang, Yaakov Bentov

**Affiliations:** 1 Toronto Centre for Advanced Reproductive Technology, M5X 2 S9, Toronto, ON, Canada; 2 Division of Reproductive Sciences, Department of Obstetrics and Gynecology, University of Toronto, Toronto, ON, Canada; 3 Samuel Lunenfeld Research Institute, Mount Sinai Hospital, Toronto, ON, Canada

**Keywords:** Low dose hCG, *In vitro* fertilization, Ovarian stimulation, Oocyte maturation, Cost of treatment

## Abstract

**Background:**

To compare the outcome of IVF cycles in women receiving controlled ovarian stimulation with recFSH or recFSH plus low dose hCG.

**Methods:**

A retrospective case control study, performed at a private practice affiliated with an academic institute. Patients were infertile women who were treated with IVF/ICSI and controlled ovarian stimulation in a long GnRH agonist protocol using either low dose hCG in addition to recFSH [N = 88] or recFSH alone [N = 99]. Primary outcomes were mean FSH dose, number of mature eggs, number of fertilized eggs, and serum levels of estradiol. Secondary outcomes were endometrial thickness, cycle cancellations and pregnancy rates.

**Results:**

A significant increase in number of mature and fertilized eggs was observed in women over 40 years of age using low dose hCG in addition to recFSH. The estradiol level was significantly higher on the day of hCG administration and the serum level of FSH on cycle day 7 and on the day of hCG administration were lower.

**Conclusion:**

Addition of low dose hCG to recFSH compared with recFSH alone significantly modified cycle characteristics in patients >/= 40 years and could be of potential benefit for IVF cycles in older infertile women.

## Background

Major advancements have been made in the treatment of infertility over the past three decades. Multiple trials have been conducted in order to achieve safe, effective, and low cost treatment protocol for controlled ovarian stimulation (COS) in patients undergoing IVF/ICSI. Despite these developments, the age of the female patient is still considered the most important prognostic factor for both response to COS and for pregnancy outcome [1,2].

The most important hormone in the stimulation of ovarian folliculogenesis is FSH [3]. There is controversy about the importance of luteinizing hormone (LH) in ovarian stimulation, although some studies have shown that LH may improve ovarian response, in particular for older women [4]. Another study showed that the use of recLH in GnRH antagonist cycles improved the implantation rate in older patients [5]. This beneficial effect was not seen with a microdose flare protocol [6]. The selective contribution of LH to the outcome of IVF treatment in older patients, has also been validated in a Cochrane meta-analysis [7].

LH and hCG share structural similarities and function through the same receptor [8]. The half-life of hCG is longer (2.32 days) than LH which has a half-life estimated to be about 1 hour [9]. In addition, hCG has stronger LH receptor binding affinity than LH [10] and has, therefore, been used as a substitute for LH [11]. It is administered at doses of 5.000-10.000 IU to mimic the endogenous LH surge and to achieve oocyte maturation and release of the cumulus-oocyte complex.

Drakakis at al demonstrated the superiority of hCG over recLH as an adjuvant to recFSH in the first four days of ovarian stimulation. The use of hCG resulted in a higher number of follicles, oocytes, peak estradiol and number of transferable embryos [11].

Filicori and colleagues demonstrated that low dose hCG can be used clinically to replace FSH to complete controlled ovarian stimulation in a long GnRH agonist protocol [12]. Following pituitary downregulation and several days of ovarian stimulation, recFSH was discontinued and stimulation was continued with 200 IU of hCG only. The outcome of the treatment was comparable to the control group that was treated with recFSH throughout the cycle. Similar findings were described also in GnRH antagonist cycles, in which, low dose hCG replaced recFSH in the final days of stimulation with a comparable outcome [13]. The addition of low doses of hCG to FSH in a long stimulation protocol in PCOS patients improved mature oocyte yield at lower cost and avoided OHSS, without a drop in pregnancy rates [14]. It has also been shown that the addition of hCG prior to FSH stimulation (hCG priming) may have a positive effect on oocyte maturity in controlled ovarian stimulation [15].

The aim of the present study was to evaluate the impact of low dose hCG as a supplement to recFSH for ovarian stimulation as part of a long GnRH agonist protocol in IVF patients. We examined the clinical outcome and the cost-effectiveness of this protocol in women <40 or ≥ 40 years of age.

## Methods

The study was a retrospective case–control study, conducted at the Toronto Center for Advanced Reproductive Technology, a University of Toronto affiliated private practice. The study was approved by the institutional ethics committee.

In this study we collected and analyzed data of 187 IVF/ICSI cycles conducted between January 2010 and November 2011. We allocated patients to two main groups according to the use of hCG as part of their IVF stimulation. Group 1 included 88 patients who received low dose hCG in conjunction with recFSH and Group 2 included 99 patients who received recFSH only for stimulation. Each group was subdivided by age to A (less than 40 years-old) and B (40 years or older).

Women in both groups were treated with a long GnRH agonist protocol (buserelin, Suprefact, Hoechst, Frankfurt, Germany) and received recFSH injections (GonalF, Serono, Canada or Puregon, Organon, Canada) from cycle day 3, and injection of 10.000 IU of hCG was given to all patients to trigger ovulation when the appropriate number of mature follicles was visualized. The initial dose of FSH was based on each patient’s clinical condition and her response in previous treatment cycles if available; the dose was then adjusted according to the patient response. Cycle monitoring was conducted by transvaginal ultrasound and serial hormonal profiles including FSH, LH, E2 and progesterone starting from CD3 of the stimulated cycle until the day of the hCG trigger.

In Group 1, low dose hCG (200 IU per day) was started on cycle day 3 until the day of the hCG trigger administration. Group 2 patients received recFSH injections alone starting on cycle day 3 of the stimulation cycle until the day of hCG trigger administration. Stimulation regimens were selected as per physician preference. There were no specific criteria used to determine the stimulation protocol. Oocytes were retrieved 36 h after the hCG trigger shot. All mature eggs retrieved were fertilized with either standard IVF or intracytoplasmic sperm injection (ICSI). Fertilization was checked at 18 hours post insemination. Embryo quality was assessed daily.

The number of embryos transferred was based on day of transfer, age of the patient and quality of embryos according to 2009 SART/ASRM (American Society of Reproductive Medicine) guidelines for the number of embryos transferred [16]. Luteal phase support consisted of progesterone vaginal suppositories 200 mg 3 times daily. Serum beta hCG measurement was done 14 days after egg retrieval and if positive, it was repeated 48 hours later. Clinical pregnancy was confirmed by ultrasound at 6-8 weeks of pregnancy.

We collected and analyzed the clinical data of patients in both groups including age, cause and duration of infertility, number of previous cycles and cancelled cycles and cause of cancellation. We also calculated the total dose of FSH given throughout the cycle.

The primary outcome measures were the dose of FSH needed, the number of mature eggs, the cost per mature egg, the number of fertilized eggs, and serum level of estradiol (E2). Secondary outcomes were endometrial thickness on the day of hCG administration, cycle cancellations and the pregnancy rate.

### Statistical analysis

Data were collected and tabulated using 2007 Microsoft office system - Excel version (Microsoft Corporation), expressed as mean + -SD and analyzed using student’s *t*-test.

## Results

We compared patients younger than 40 years old using low dose hCG (Group 1-A) to using recFSH (Group 2-A) and patients 40 years or older using low dose hCG (Group 1-B) to using recFSH alone (Group 2-B). Each groups had comparable baseline features at the start of the cycle including mean age, BMI, indication for treatment, method of fertilization and semen parameters and baseline hormonal profile (table 1).

**Table 1:** Baseline characteristics of patients

	**Group 1-A r FSH + Low dose hCG < 40 y N = 49**	**Group 2-A r FSH < 40 y N = 67**	**P Value**	**Group 1-B r FSH + Low dose hCG ≥ 40 y N = 39**	**Group 2-B r FSH ≥ 40 y N = 32**	**P Value**
Age (Mean ± SD) years old	35.86 ± 2.95	34.90 ± 3.23		42.00 ± 1.02	41.57 ± 0.99	
BMI (Kgm2)	23.81 ± 5.23	23.02 ± 4.31	0.508	23.0 ± 3.69	22.88 ± 3.28	0.901
**Cause of infertility**						
Ovarian	3	6				
Tubal	16	23				
Male Factor	17	21				
Endometriosis	2	3				
Others	3	4				
LMA				23	19	
LMA + Male factor				9	6	
LMA + Endometriosis				3	2	
LMA + Tubal				4	5	
**Semen Concentration**	27.36 ± 23.19	27.12 ± 28.40	0.481	28.82 ± 28.26	26.04 ± 23.75	0.333
**Insemination Method**						
IVF	14	11		6	5	
ICSI	27	48		32	22	
IVF/IVSI	4	6				

The number of cancelled cycles was almost the same in both groups. There were three cancelled cycles in group 1-A, two because of poor response and one at patient request. There were two cancelled cycles in group 2-A because of poor response. There were no cancelled cycles in group 1-B and four cancelled cycles in group 2-B because of poor response. Patients were considered to be poor responders if less than three follicles developed.

We found no significant differences in the hormonal profiles on any cycle day (Table 2), total dose of FSH used, number of mature eggs, and number of fertilized eggs in the women under the age of 40 years (Table 3). In group1-A, there were no cases of ovarian hyper-stimulation syndrome (OHSS) compared to 5 cases recorded in group 2-A. All cases of OHSS were mild to moderate and no embryo transfer was done in these cycles.

**Table 2:** Hormonal profile and ultrasound measurements in treatment groups

	**Group 1-A r FSH + Low dose hCG < 40 y N = 49**	**Group 2-A r FSH < 40 y N = 67**	**P Value**	**Group 1-B r FSH + Low dose hCG ≥ 40 y N = 39**	**Group 2-B r FSH ≥ 40 y N = 32**	**P Value**
**CD3 FSH (IU/L)**	5.38 ± 2.61	5.78 ± 3.26	0.243	6.97 ± 2.1	7.42 ± 2.78	0.232
**CD3 LH (IU/L)**	3.55 ± 2.61	3.67 ± 0.58	0.19	3.63 ± 2.02	4.37 ± 2.85	0.119
**CD3 E2 (pmol/L)**	112.23 ± 131.99	114.73 ± 51.89	0.451	120.39 ± 105.46	123.84 ± 37.04	0.426
**CD 3 Progesterone (nmol/L)**	4.13 ± 2.42	3.33 ± 2.23	0.063	4.21 ± 2.07	5.57 ± 4.48	0.066
**FSH (IU/ML) on day of HCG admin**	13.35 ± 5.14	14.38 ± 6.35	0.486	17.38 ± 4.93	19.96 ± 5.83	0.037
**Estradiol (pmol/L) on day of HCG admin**	8354.51 ± 4171.09	8226.81 ± 5262.10	0.443	8692.51 ±4174.06	6858.54 ± 3461.78	0.027
**Estradiol (pmol/L) at HCG admin/Mature egg**	1296.84 ± 1154.87	935.25 ± 436.13	0.025	1933.70 ± 1475.24	2214.41 ± 1946.52	0.265
**Progesterone (nmol/L) on day of HCG admin**	8.54 ± 4.23	6.87 ± 3.49	0.016	7.59 ± 3.88	6.48 ± 2.38	0.081
**Endometrial thickness (mm) on day of HCG admin**	1.02 ± 0.27	1.04 ± 0.25	0.336	0.98 ± 0.25	0.921 ± .026	0.125

Both groups were comparable with regard to their baseline hormonal profile.

**Table 3:** Cycle outcome measures

	**Group 1-A r FSH + Low dose hCG < 40 y N = 49**	**Group 2-A r FSH < 40 y N = 67**	**P Value**	**Group 1-B r FSH + Low dose hCG ≥ 40 y N = 39**	**Group 2-B r FSH ≥ 40 y N = 32**	**P Value**
**Starting dose of FSH**	246.43 **± 61.45**	238.06 **± 71.96**	0.501	302.56 **± 41.28**	322.65 **± 60.03**	0.114
**Total dose of FSH.**	2,496 **± 774**	2,284 **± 843**	0.082	3,217 **± 874**	3,264 **± 1,009**	0.415
**Treatment duration, days**	10.02 **± 1.73**	9.45 **± 1.48**	0.064	10.03 **± 1.58**	9.40 **± 1.90**	0.072
**Need for dose change**	8/94 (8.5%)	12/67 (17.9%)	0.412	5/39 (12.8%)	4/32 (12.5%)	0.193
**No of retrieved oocytes**	10.83 **± 6.55**	12.50 **± 7.29**	0.10	7.92 **± 4.32**	6.28 **± 4.50**	0.067
**No of mature eggs**	9.49 **± 5.78**	10.37 **± 6.55**	0.230	5.72 **± 3.52**	4.00 **± 3.34**	0.019
**No of fertilized eggs**	6.71 ± 4.43	7.35 ± 4.35	0.227	4.89 ± 2.74	3.70 ± 2.09	0.027
**Fertilization rate per retrieved oocyte**	61.47% ± 18.38	62.97% ± 21.39	0.347	58.96% ± 19.48	58.72% ± 33.48	0.394
**No of Embryos per transfer**	2.66 ± 1.22	2.68 ± 1.16	0.459	2.92 ± 1.09	3.00 ± 1.37	0.399
**No of frozen embryos**	2.90 ± 3.66	4.14 ± 4.62	0.128	1.03 ± 2.13	0.296 ± 0.95	0.033
**Total pregnancies per cycle started**	12/49 (24.49%)	23/67 (34.33%)	0.416	6/39 (15.38%)	3/32 (9.38%)	0.090
**Total pregnancies per cycle completed**	12/45 (26.67%)	23/60 (38.33%)	0.152	6/38 (15.79%)	3/27 (11.11%)	0.588
**Cancelled cycles**	3	2		0	4	
**Hyper stimulation**	0	5		0	0	
**No eggs retrieved**	1	0		1	1	

In contrast, when comparing the older women (groups 1-B and 2-B), we found that E2 levels on the day of hCG administration were significantly higher while FSH levels were lower on cycle day 7 (P = 0.027) and on day of hCG administration (P = 0.037) in the group that used low dose hCG [group 1-B]. When compared with group 2-B, the number of mature eggs was higher (P = 0.019), the number of fertilized eggs was higher (P = 0.027) and the number of frozen embryos (0.033) was higher in group 1-B. (Table 3)

Overall, there were no differences in endometrial thickness or fertilization rates in any of the groups. Pregnancy rates were not significantly different between group 1-A and 2-A, and between 1-B and 2-B.

### Cost analysis

The cost was determined using the average cost of a single unit of recFSH which was approximately Canadian $1.00 during this study. The cost-effectiveness ratio was calculated by determining the average cost of the stimulation protocol for each mature oocyte. The overall cost of medications per cycle started was almost the same but the cost/mature follicle was less in the low dose hCG group in the women > 40 years of age (P = 0.053). The average cost/mature follicle in group (1-B) was $878.78 while in the FSH only group (2-B) it was $1493.17.

## Discussion

In this study we evaluated the effects of adding low dose hCG to recFSH for ovarian stimulation in women undergoing IVF. We compared the outcome of this protocol with patients of the same age who received recFSH alone. The results showed that the combination of low dose hCG and recFSH improved the outcome of IVF cycles in the advanced age group by increasing the number of mature oocytes, the number of fertilized eggs and reduced the cost/mature egg. We did not detect a significant effect of addition of low dose hCG to the group of younger women. Previous prospective randomized studies showed that the addition of recLH to recFSH improved the implantation and pregnancy rates among older patients undergoing a long GnRH-agonist down regulation protocol, in comparison to younger patients. Therefore our finding that the older patients seem to benefit more from the addition of hCG is consistent with previous reports on recLH [4,17].

In natural cycles, follicular maturation and development is a long, complex process beginning months before ovulation of a mature oocyte [18,19]. According to the two cell theory, both FSH and LH are necessary for folliculogenesis and ovulation. LH promotes the production of androgens from cholesterol and pregnenolone by stimulating 17-alpha-hydroxylase activity in theca cells. Androgen diffuses to the granulosa cells where FSH induces the expression of aromatase [20] to enable conversion of androgens to estrogen [21] . In addition FSH induces LH receptors on the granulosa cells of developing antral and preovulatory follicles [22].

For a selection of a single dominant follicle, LH pulses are required for maintenance of theca enzymes to produce androgen substrate for aromatization, and LH may also support the growth and maturation of larger follicles through binding to specific granulosa cell LH receptors that develop after FSH priming [23,24]. In a long GnRH agonist protocol, LH secretion is suppressed through pituitary downregulation. The addition of daily low dose hCG to recFSH during ovarian stimulation may provide sufficient amounts of LH activity [25] to improve treatment outcomes for patients with biologically older ovaries [26].

In a long GnRH agonist cycle, the lowest serum concentration of LH will be reached in the late follicular phase. At this stage of folliculogenesis in natural cycles, the mature follicles gradually transition their control to LH by increasing the number of granulosa cell LH receptors. Hence, several authors suggested that the late follicular phase is the most appropriate time to add LH/hCG to the stimulation as successfully reported by Filicori et all [12]. On the other hand, hCG priming was shown to increase testosterone production [27] which in turn, will induce an increased expression of follicular FSH receptors and greater sensitivity to stimulation. The administration of hCG in the first four days of ovarian stimulation resulted in a significant increase in the number of mature follicles, retrieved eggs, implantation and pregnancy rate [11]. In the present study we decided to combine the two approaches and provide hCG throughout the FSH ovarian stimulation.

In none of the cycles included in the study did low dose hCG administration cause premature luteinization. Also, similar to our experience, an earlier study by Filicori and colleagues showed that 200 IU of hCG is well tolerated with no adverse effects detected in patients receiving this treatment [11].

The present study showed that serum FSH levels were significantly lower on cycle day 7 and on the day of hCG administration in the advanced age group that received low dose hCG. A possible explanation for this observation might be the increased production of androgen by theca cells under the effect of low dose hCG which in turn increases the sensitivity of granulosa cells to utilize FSH [23].

Despite the lack of a significant difference in pregnancy rates between the hCG and control groups, the increased number of frozen embryos in the older patients receiving hCG (group 1-B) could potentially translate to a higher overall cumulative pregnancy rate [28] .

We also have to mention that no cases of ovarian hyperstimulation syndrome were recorded in patients who received low dose hCG in addition to recFSH while 5 cases were recorded in recFSH only group. The same was observed in other studies [29]. Potential explanations include the promotion of growth of larger, mature follicles only by the hCG and a decreased production of endothelial growth factor (VEGF), which tends to be secreted from the relatively smaller follicles [30]. VEGF is known as an important factor in development of OHSS.

The main limitation of this study was its retrospective design with the inherent problems related to selection bias in the patients assigned to each treatment. While we cannot correct for this problem, our data analysis suggests that the patients in group 1 and group 2 were comparable in all demographic parameters including age and baseline FSH levels. We believe that our findings provide preliminary evidence of a beneficial effect of low dose hCG supplementation in recFSH stimulation in older women undergoing IVF treatment, and should serve as a basis for a future prospective randomized controlled trial.

## Conclusion

The addition of hCG to recFSH throughout the stimulation in a long GnRH IVF protocol had only benefited the group of patients 40 years and older. The recorded benefits included a higher number of mature and fertilized eggs and frozen embryos. While the cost per mature egg was lower.

## Abbreviations

ASRM, American Society of Reproductive Medicine; COS, Controlled ovarian stimulation; E2, Estradiol; FSH, Follicular stimulating hormone; GnRH, Gonadotropin realising hormone; hCH, Human chorionic gonadotropin; ICSI, Intra-cytoplasmic injection; IU, International units; IVF, In vitro fertilization; LH, Luteinizing hormone; OHSS, Ovarian hyper-stimulation syndrome; recFSH, Recombinant FSH; recLH, Recombinant LH; SART, Society for Assisted Reproductive Technologies; VEGF, Vasoendothelial growth factor.

## Competing interests

The authors declare that they have no competing interests.

## Author’s contributions

HG collected, analyzed the data and wrote the manuscript. RFC, NE, PC, YB supervised the study and revised the manuscript. All authors read and approved the final revision of manuscript.
